# Investigating Germ Cell Transition Genes in Breast Cancer: Exploring the Genesis of Cancer Testis-Associated Markers

**DOI:** 10.3390/ijms26188958

**Published:** 2025-09-15

**Authors:** Hamid Khodayari, Saeed Khodayari, Mohammad Dashtkoohi, Amirnader Emami Razavi, Seyed Rouhollah Miri, Ahad Mohamadnejad, Marcelo de Castro Santos, Fabio Comuzzi, Reza Shirkoohi, Habibollah Mahmoodzadeh, Karim Nayernia

**Affiliations:** 1 Cancer Research Center, Cancer Institute of Iran, Tehran University of Medical Sciences, Tehran 1419733141, Iran; h.khodayari@hotmail.com (H.K.); saeed.khodayari@emory.edu (S.K.); md1999c@gmail.com (M.D.); drsrmiri@gmail.com (S.R.M.); rshirkoohi@tums.ac.ir (R.S.); 2 International Center for Personalized Medicine (P7MEDICINE), Luise-Rainer-Str. 6-12, 40235 Düsseldorf, Germany; 3 Iran National Tumor Bank, Cancer Institute of Iran, Tehran University of Medical Sciences, Tehran 1419733141, Iran; razavinader@gmail.com; 4 Cancer Biology Research Center, Cancer Institute, Tehran University of Medical Sciences, Tehran 1419733141, Iran; ahad.mohammadnejad@yahoo.com; 5 Sanacare Gruppenpraxis Rue du Marche-Neuf 27, 2503 Bienne, Switzerland; marcelo.decastrosantos@sanacare.ch; 6 Hospital Du Jura, Faubourg Capucin 30, 2800 Delémont, Switzerland; fabio.comuzzi@h-ju.ch

**Keywords:** breast cancer, epithelial-to-germline transition, cellular clustering, in silico analysis, single-cell RNA sequencing, observational study

## Abstract

Transition is an essential mechanism that drives the development of distinct cellular phenotypes and tumorigenesis. The expression of various types of testis cancer antigens (TCAs) in breast carcinomas suggests a potential transition to male germ cell features within the tumor. This study explores the cellular populations in breast cancer that express genes associated with male germ cell development. We re-analyzed published datasets to identify the germline-associated genes in breast tumors. We then experimentally validated the expression of the identified genes in 28 breast tissue tumor samples using a quantitative RT-PCR. Based on available datasets, we also performed single-cell RNA sequencing (scRNA-seq) to analyze the tumor heterogeneity and cellular clustering. A total of 455 overexpressed genes were identified that were related to fetal primordial germ cells (PGCs), particularly those in the male gonad. Our examinations showed a significant overexpression of five genes (*CCNB1*, *CCNB2*, *PTTG1*, *RACGAP1*, and *UBE2C*) in the tumor samples. The scRNA-seq analysis revealed 14 distinct cell clusters, characterized by different gene expression signatures and cell cycle phases. The breast tumor stromal cells were suggested as the main source of the germline-associated genes. This study provides insights into the molecular mechanisms and pathways involved in germ cell transition in breast carcinoma.

## 1. Introduction

Breast cancer, as identified in the latest GLOBOCAN study, remains the second most commonly diagnosed malignancy globally across both sexes [1]. The complexity in addressing breast carcinoma is due to a multitude of factors, including the intra-tumoral heterogeneity of the cells with different potentials of cellular proliferation, invasiveness, variable responses to treatments, and frequent genetic mutations. Despite extensive research, the underlying biological processes that drive the development of carcinoma cells and their heterogenicity are yet to be completely understood [2].

The concept of the epithelial-to-mesenchymal transition (EMT) has typically been accepted in the definition of carcinoma development and progression [3]. The EMT, a process essential for normal fetal development, organogenesis, and tissue repair, involves the transformation of epithelial cells into mesenchymal cells, enhancing their movement and differentiation capabilities [3,4]. This transition, required during embryogenesis in the epiblast cell layer, is instrumental in the formation of the three germ layers among the gastrulation. In adults, however, the dysregulation of this process can influence cancer cells’ development, metastatic potential, and aggressiveness [4,5]. The pathological EMT could particularly be promoted through the altered activation of signaling pathways, like transforming growth factor beta (TGF-β), the wingless-related integration site (Wnt), Notch, and Hedgehog. Additionally, the EMT contributes to various cancer hallmarks, including stemness, resistance to apoptosis, angiogenesis, and immune evasion [4,5].

Recent studies have brought to light the possible role of germline-associated genes, like testis cancer antigens (TCAs), in the development of breast carcinoma [6]. These antigens are typically restricted to the testis in normal postnatal life, although aberrantly expressed TCAs have been observed in various cancers [6,7]. In addition, the active expression of some testis-specific genes, including *testis specific-10* (*TSGA-10*), *testis expressed-101* (*TEX101*), and the *outer dense fiber of sperm tails-3* (*ODF-3*), has already been approved in breast cancer as the cancer–testis (CT) gene [8]. Similarly, an experimental study highlighted the vital role of another CT gene, named the *Piwi-like RNA-mediated gene silencing-2* (*Piwil-2*) factor, in breast cancer stem cells (BCSCs) [9,10]. The *Piwil2* gene, which is a member of the PIWI protein family, could influence the development and differentiation of germline stem cells (GSCs). This factor could interact with a range of genes and notably participates in suppressing transposable elements, DNA sequences capable of moving within the genome and causing mutations [9,10,11]. Correlations have been observed between *Piwil-2* expression and the proliferation and survival of BCSCs. Notably, increased *Piwil2* activity seems to restrict the proliferation and survival of BCSCs through the Stat3/Bcl-XL pathway [12]. The expression and functional activation of these CT genes could suggest a mechanism implicating the germline-like cells’ development in breast tumors. Furthermore, the cellular clusters expressing the germline-associated genes would be considered as the main population presenting TCAs.

Building on this premise, our observational study proposes a possible mechanism in breast tumors characterized by cells with germline traits. This mechanism, termed the epithelial-to-germline transition (EGT), is hypothesized as a possible transformative process leading to the formation of germline-like cells into the tumor. Our research aims to identify and analyze genes involved in this transition in breast carcinoma and to explore their impact on the tumor microenvironment and cellular heterogeneity.

## 2. Results

### 2.1. Bioinformatics Analysis of Breast Tumor Differential Gene Expression

**Figure 1:**
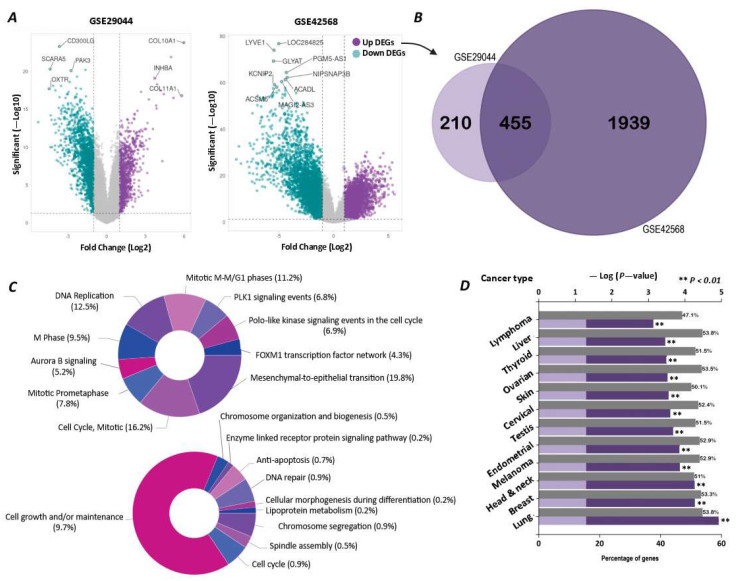
Differential gene expression analysis of breast tumors compared to healthy breast tissue. (**A**) Volcano plots for two studies, GSE29044 and GSE42568, showing the log fold change and statistical significance of differentially expressed mRNAs. (**B**) Venn diagram of genes with increased expression pression at Fc ≥ 1, with a significance coefficient of *p*-value ≤ 0.05 in both studies. (**C**) Pathway enrichment analysis (upper pie chart) and biological process enrichment analysis (lower pie chart) of the 455 common genes between the two studies. Since individual genes may be involved in multiple categories, the total sum exceeds 100%. (**D**) Bar chart of cancer type enrichment analysis for the 455 upregulated genes, showing the key tumors associated with them.

We performed a series of bioinformatics analyses on the differential gene expression of breast tumors using the available data from two GSE29044 and GSE29431 studies. The analysis of the GSE29044 study demonstrated that 9472 mRNAs had a significantly altered expression compared with the normal tissue, of which 883 genes had an increased expression with LogFc ≥ 1. In the case of the GSE29431 study, 5813 mRNAs had obtained significantly altered expressed genes compared with the normal tissue. In this regard, 1086 genes had been upregulated with LogFc ≥ 1 (Figure 1A). The outcome of our Venn analysis demonstrated that 455 genes were commonly upregulated in both datasets, with LogFc ≥ 1 (Figure 1B) (raw data is available in a Supplementary Excel file). The enrichment analysis of 455 shared upregulated genes indicated that these genes were mainly involved in some cellular processes, including proliferation, mitosis, and differentiation (Figure 1C). In addition, the cancer type enrichment analysis of the above genes showed that they played a crucial role in the carcinogenesis of several malignancies, like lung cancer, breast cancer, head and neck cancer, and melanoma (Figure 1D).

### 2.2. Association of Breast Tumors’ Differentially Expressed Genes with PGCs

**Figure 2:**
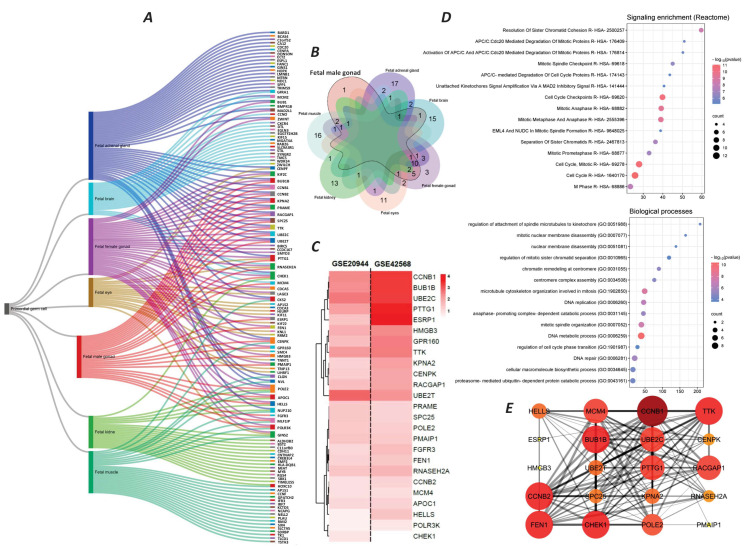
Identification of germ cell types associated with 455 genes and the function of target genes. (**A**) Ribbon diagram of germ cell types detected by cell type enrichment analysis. (**B**) Venn diagram of genes associated with each germ cell type, with a focus on fetal male gonad cells. (**C**) Heatmap diagram of expression levels (Log2Fc) of 25 genes related to fetal male gonad cells in two studies, GSE29044 and GSE42568. (**D**) Pathway enrichment analysis (upper pie chart) and biological process enrichment analysis (lower pie chart) of 25 genes related to fetal male gonad cells. (**E**) PPI analysis of 25 genes related to fetal male gonad cells. Larger and more intensely colored nodes represent proteins with the most interactions. The thickness of the lines indicates the confidence score of the interaction.

The cell type annotation revealed that our 455 target genes in breast tumors were associated with seven types of PGCs. These cell types included fetal adrenal gland PGCs, fetal female gonad PGCs, fetal muscle PGCs, fetal male gonad PGCs, fetal brain PGCs, fetal kidney PGCs, and fetal eye PGCs. The number of active genes in each cell type was 42, 35, 26, 25, 20, 20, and 18, respectively (Figure 2A). The relationships and overlaps of mRNAs in each cell type are shown in the ribbon and Venn diagrams in Figure 2A,2B. Our analysis remarked that the genes present in fetal male gonad PGCs had the most overlap with other cell types (Figure 2B). The enrichment analysis on the 25 active genes in fetal male gonad PGCs also showed that this group of mRNAs is mainly involved in mitosis and molecular processes related to cell division (Figure 2C,D). The protein–protein interaction (PPI) analysis of the 25 target genes also revealed that 11 mRNAs, including BUB1 mitotic checkpoint serine/threonine kinase B (BUB1B), Cyclin B1 (CCNB1), Cyclin B2 (CCNB2), checkpoint kinase 1 (CHEK1), flap structure-specific endonuclease1 (FEN1), minichromosome maintenance complex component 4 (MCM4), DNA polymerase epsilon subunit 2 (POLE2), pituitary tumor transforming gene 1 (PTTG1), Rac GTPase activating protein 1 (RACGAP1), TTK protein kinase (TTK), and ubiquitin-conjugating enzyme E2 C (UBE2C), act as the possible key players in these networks (Figure 2E). These genes were selected based on a PPI network analysis using the STRING database, and the prioritization was performed based on the degree centrality, where genes with a degree score greater than 14 were considered to have a higher network connectivity and biological relevance.

**Figure 3:**
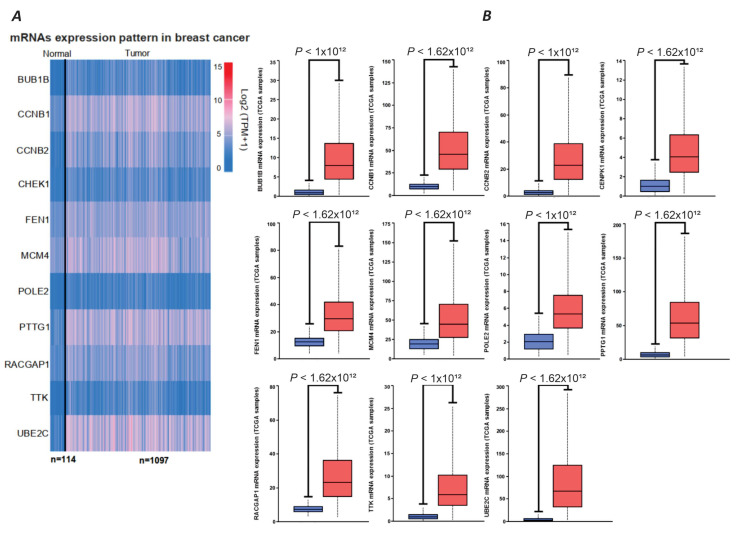
Expression levels of 11 key mRNAs active in the PPI network of fetal male gonad cells and breast cancer, based on TCGA database results. (**A**) Heatmap of the expression levels of the 11 genes in tumor tissue and normal breast tissue. (**B**) Box plot of the expression levels of the 11 genes in tumor tissue (1097 samples) and normal breast tissue (114 samples).

The analysis of genomic data from “The Cancer Genome Atlas Program (TCGA)” database, https://ualcan.path.uab.edu/analysis.html (accessed on 20 July 2021), on 1097 breast cancer tumor samples displayed a significant and noticeable difference in the expression level of all the above-identified 11 genes compared to the normal breast tissues (Figure 3A). A heatmap of the 11 mRNA expression levels of the tumor samples displayed that the seven genes, including *CCNB1*, *CCNB2*, *CHEK1*, *FEN1*, *PTTG1*, *RACGAP1*, and *UBE2C*, had higher expression levels in the tumor tissue (Figure 3B). Therefore, based on our bioinformatic analysis, we have identified all seven genes as potential PGC-associated genes in breast cancer for further experimental investigation regarding their expression in breast tumor samples.

### 2.3. Relative Gene Expression Analysis of PGC-Associated Genes in Breast Tumors

**Figure 4:**
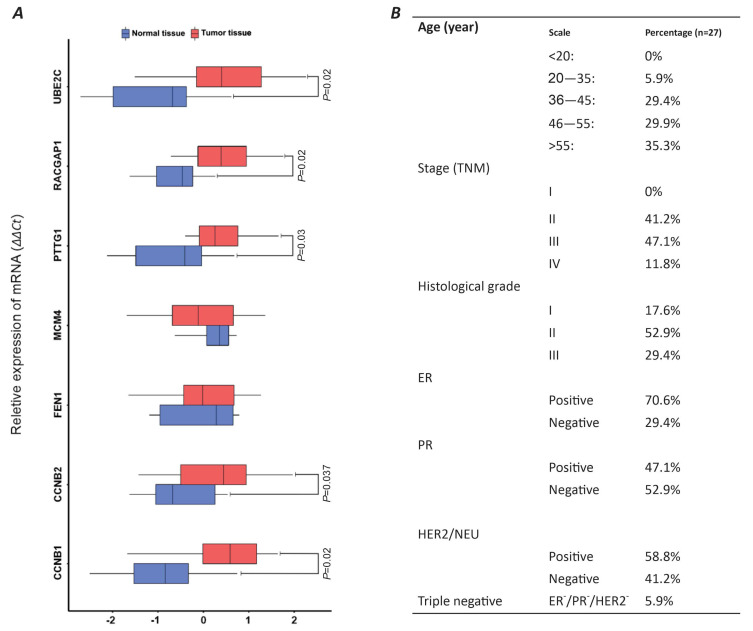
Comparative analysis of panel 11 genes across 27 breast tumor samples using qRT-PCR analysis. (**A**) BoxPlot illustrating relative gene expression levels in tumor tissue versus healthy breast tissue via 2∆∆Ct calculation. (**B**) Patient demographics and tumor phenotypic characteristics.

In this study, we relatively assess the expression of our candidate genes in the tumor samples of 27 individuals with breast carcinoma. The majority of the patients (55%) were over 55 years old, while 29.4% were in the 36–45 and 46–55 age groups, respectively. Only 5.9% of the patients were in the 20–35 age range. No patients under 20 years old were included in this study. The most common stage of cancer among the patients was stage 3 (47.1%), followed by stage 2 (41.2%). Only 11.8% of the patients had metastatic breast cancer. There were no cases of stage 1 breast cancer in the study (Figure 4B). Based on the histological grading of tumors, more than half of the tumors (52.9%) were GII, while 29.4% were GIII, and only 17.6% were diagnosed as GI. The prevalence of tumors with positive estrogen receptor (ER), progesterone receptor (PR), and human epidermal growth factor receptor 2 (HER2) phenotypes in the study was 70.6%, 47.1%, and 58.8%, respectively. Only 5.9% of the tumors were of the triple-negative breast tumor type (Figure 4B).

The relative gene expression analysis of seven PGC-associated genes, including *CCNB1*, *CCNB2*, *FEN1*, *MCM4*, *PTTG1*, *RACGAP1*, and *UBE2C*, was conducted on the samples of breast tumors compared to healthy mammary tissues. The result of our experience has highlighted a significant overexpression of five *CCNB1*, *CCNB2*, *PTTG1*, *RACGAP1*, and *UBE2C* genes in breast tumors compared to the normal tissues (*p*-value < 0.05) (Figure 4A). The average expressions of these genes in breast tumor samples compared to healthy mammary biopsies (tumor/healthy) were as follows: CCNB2 (0.483/−0.869), CCNB1 (0.262/−0.472), PTTG1 (0.398/−0.719), RACGAP1 (0.338/−0.609), and UBE2C (0.565/−1.016). There was no significant difference in the relative expression of FEN1 and MCM4 between the tumors and healthy tissue (Figure 4A).

### 2.4. Breast Tumor Cellular Clustering

**Figure 5:**
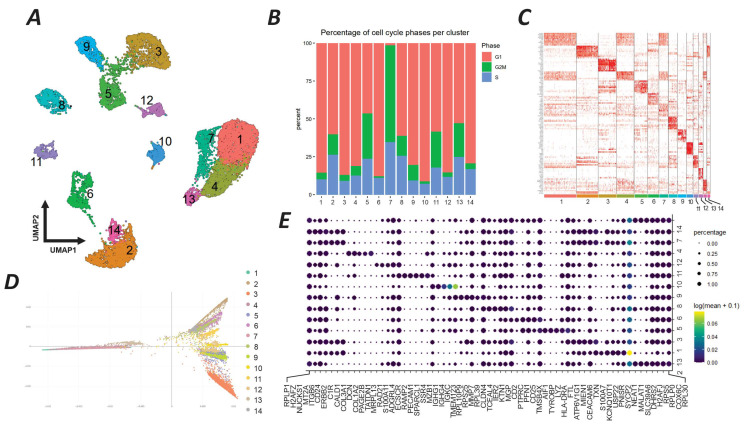
scRNA-seq analysis of breast tumor cells from the GSE180286 study. (**A**) UMAP plot showing 14 distinct clusters (1–14) of cells based on their single-cell transcriptomic data. Each cluster has a unique color and number for reference. (**B**) Stacked bar chart showing the percentage of cells in different phases of the cell cycle (G1, S, G2/M) within each cluster. The color coding for the cell cycle phases is as follows: red for S phase, green for G1 phase, and blue for G2/M phase. (**C**) Heatmap of the top 10 genes with high expression in each cluster. The intensity of red color indicates higher expression levels, providing a visual representation of the gene expression landscape across the clusters. (**D**) Diffusion map generated by the destiny package, displaying the developmental trajectory of each cluster. This map visualizes the probable transition states of cells according to their developmental processes, with colors corresponding to the clusters defined in the UMAP plot. (**E**) Dot plot illustrating the key genes regulating the developmental trajectory of the clusters as analyzed by Monocle 3. The size of the dots represents the percentage of cells expressing the gene in the cluster, while the color intensity indicates the expression level (log2 fold change).

Based on the results derived from our experimental evaluations, a series of scRNA-seq analyses on the GSE180286 breast tumor dataset were performed for the characterization of the tumor microenvironment and cellular clustering. In this regard, we have identified 14 separate clusters of cells, each exhibiting distinct transcriptomic signatures, as depicted in the UMAP plot (Figure 5A). The clusters represent different cellular states within the tumor. We first analyzed the cell cycle phases of each cluster and found three separate clusters of cells. Clusters 2, 7, 8, and 13 had a higher percentage of cells in the S phase, indicating a high proliferation status. Clusters 1, 3, 6, and 10 mostly had cells in the G1 phase, representing an intermediate proliferative state. Clusters 5, 7, and 11 had a greater proportion of cells in the G2/M phase (Figure 5B). The heatmap shows the expression pattern of each cluster, with marked differences in the top 10 active genes, indicating the diverse functional states across the tumor cell populations (Figure 5C). The destiny package diffusion map reveals developmental trajectories within the tumor microenvironment (Figure 5D). Clusters 1, 4, and 7 were closely aligned, suggesting a shared developmental pathway or a sequential progression in cellular differentiation. Clusters 2, 6, 8, and 14 were dispersed, indicating divergent developmental trajectories (Figure 5D). The dot plot analysis (Figure 5E) identifies key genes with significant regulatory potential within each cluster. Cluster 3 had a pronounced activity of genes such as the *SRY-box transcription factor 4* (*SOX4*) and *epidermal growth factor receptor* (*EGFR*), which may influence its distinct developmental pathway. Similarly, cluster 7 was characterized by the active expression of *Notch receptor 1* (*NOTCH1*) and the *aldehyde dehydrogenase 1 family member A1* (*ALDH1A1*) genes, suggesting a role in the regulation of its unique cellular phenotype.

**Figure 6:**
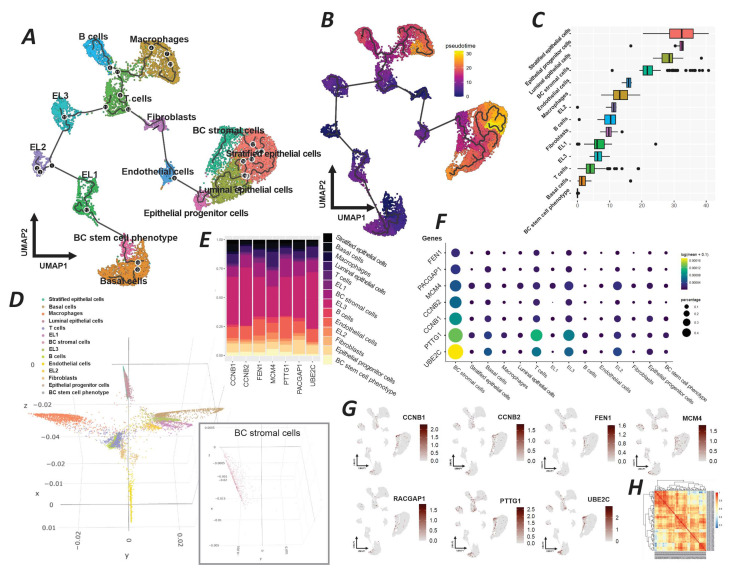
Comprehensive single-cell RNA sequencing analysis of breast cancer cellular heterogeneity and gene expression dynamics. (**A**) UMAP projection with trajectory analysis illustrating the differentiation paths of various cell types within the breast cancer microenvironment, including basal cells, breast cancer stem cell phenotype, and differentiated epithelial cells. (**B**) UMAP overlay with pseudotime analysis indicating the developmental progression of cells from progenitor to differentiated states. (**C**) Box plot generated by the destiny package, illustrating the distribution of cellular states along a developmental continuum, revealing the diversity in cell type maturation. (**D**) A 3D diffusion map highlighting the developmental trajectories of cell populations, with an inset showing the specific localization of breast cancer stromal cells. (**E**) Stacked bar chart representing the proportional gene expression levels of target genes associated with germline cells across different clusters. (**F**) Dot plot visualizing the expression levels and prevalence of specific target genes within each cluster, with dot size representing expression percentage and color intensity indicating expression level (log2 fold change). (**G**) Feature plots from Seurat pinpointing the clusters with predominant expression of target genes, showcasing the specific cellular niches where these genes are most active. (**H**) Heatmap of correlative gene expression active in the breast tumor stromal cells cluster, illustrating the interplay and potential regulatory relationships between genes.

The UMAP analysis of our experimentally approved PGC-associated genes delineated distinct cell populations within the breast cancer tissue, such as B cells, T cells, macrophages, fibroblasts, endothelial cells, luminal epithelial cells, and several others. The trajectory analysis illustrates differentiation pathways among various cell types with breast tumor stromal cells, epithelial progenitor cells, and basal cells charting distinct routes, showing diverse origins and lineage commitments (Figure 6A).

The pseudotime overlay further quantifies this developmental progression, situating breast cancer stromal cells along a continuum that suggests a maturation from a less differentiated state toward a more specialized stromal phenotype (Figure 6B). A detailed box plot derived from the destiny package analysis highlights the distribution of the pseudotime across different cell types, with breast tumor stromal cells exhibiting a broad range of states, proposing a spectrum of maturation within this group (Figure 6C). This variability underscores the dynamic nature of the tumor stroma and its potential impact on tumor biology. The 3D diffusion map projects the complexity of cellular trajectories in a spatial context, which reveals the intricate developmental paths undertaken by the cells (Figure 6D). Specifically, the breast tumor stromal cell cluster is indicated, showing its distinct spatial domain within the tumor architecture. This visualization emphasizes the unique trajectory of tumor stromal cells, potentially reflective of their functional specialization. The stacked bar chart elucidates the expression levels of target genes associated with germline cells across different clusters (Figure 6E). Breast tumor stromal cells exhibit a distinct expression profile, marked by the expression of genes such as *FEN1*, *RACGAP1*, and *MCM4*, which may delineate their functional identity. The dot plot provides a granular view of the expression patterns of these target genes, with stromal cells demonstrating a notable expression of genes such as *CCNB1*, *CCNB*, and *UBE2C* (Figure 6F). The size of the dots corresponds to the proportion of cells expressing the gene, while the color intensity indicates the expression strength, signifying the gene’s regulatory potential within this cluster. Feature plots from Seurat further refine this analysis by showing the clusters with a predominant expression of the target genes (Figure 6G). The stromal cells of breast tumors show a marked expression of genes such as *PTTG1* and *RACGAP1*. It highlights the cluster’s potential role in the tumor environment. Finally, the correlation heatmap presents the inter-gene expression relationships within breast tumor stromal cells, revealing a network of potentially interacting genes (Figure 6H).

## 3. Discussion

Our results are consistent with previous studies that have reported the aberrant expression of testis cancer antigens in various malignancies, including breast cancer. TCAs, also known as cancer–testis antigens (CTAs), are a group of proteins that are normally expressed only in the testis and the germlines but are reactivated in malignant cells [6,7]. The CTAs’ activity has been implicated in tumorigenesis, metastasis, immune evasion, and resistance to therapy. From the developmental view, a trophoblast model of the tumorigenesis could be suggested for the explanation of CTAs’ expression in breast tumors [13]. In this model, a similar process of embryogenesis, spermatogenesis, and carcinogenesis were suggested at the molecular level for cancer development. This theory gained more evidence when it was discovered that human cancers frequently produce chorionic gonadotropin and other trophoblastic paracrine factors, indicating a link between germ cell development and cancer progression [13,14]. As a result, scientists identified a growing number of CTAs—proteins exclusively expressed in trophoblasts, germ cells, and tumor cells. Janic et al. (2010), in a drosophila model, have proven that the ectopic expression of CTAs could actively lead to the formation of malignant brain tumors [15]. And the CTAs’ suppression was ultimately inhibiting the tumor growth [15]. The above-mentioned results have supported the fact that the reactivation of the gametogenesis and PGC pathways in normal cells could ultimately lead to tumorigenesis. Moreover, CTAs have been shown to be associated with stemness, a property of cancer cells that enables them to self-renew and differentiate into various cell types [16,17]. It has been believed that CTAs may play a crucial role in driving several cellular pathways that lead to the formation of cancer cells and the reactivation of critical hallmarks of cancer [18]. However, there is no clear explanation or evidence about the cellular pathways or upstream genes that promote the activation process of CTAs, particularly in breast cancer. To shed light on this matter, our study hypothesized that by changing the differentiation status of cells through a transition phenomenon, the activation of certain specific genes of PGCs in breast cancer tumors could lead to the appearance of CTAs. This study suggests that the population of the breast carcinoma cells may exploit the mechanisms of the PGC development, like the EMT, to acquire germline traits and stemness, thereby enhancing their malignant potential. This hypothesis was first supported by our in silico analysis. We have managed to identify a panel of 11 genes in the breast tumors as the key players in the networks of fetal male gonad PGC development. These genes include *BUB1B*, *CCNB1*, *CCNB2*, *CHEK1*, *FEN1*, *MCM4*, *POLE2*, *PTTG1*, *RACGAP1*, *TTK*, and *UBE2C,* which were also involved in some cellular processes, such as proliferation, mitosis, and cell division. Furthermore, this panel was known to regulate the cell cycle, DNA replication, DNA repair, and chromosome segregation and to be associated with genomic instability.

Previous research has suggested that the aberrant expression of TCA genes may reflect a form of developmental plasticity, whereby epithelial tumor cells temporarily take on germline-like characteristics through epigenetic and transcriptional reprogramming [13]. This process may help tumor cells escape normal regulatory constraints and gain properties linked to stemness, immune evasion, and therapeutic resistance [15]. Gaining a clearer understanding of the EGT in breast cancer could therefore shed light on new drivers of disease progression and reveal opportunities for targeted therapy. While the EMT is a well-recognized contributor to invasion and metastasis, recent evidence indicates that EMT-like intermediate states may come before the EGT and help to set the stage for it. These transient mesenchymal states may allow for chromatin rearrangement and transcriptional rewiring, ultimately resulting in the acquisition of germline-like traits. Taken together, this points to a model in which the EGT may function downstream of, or in tandem with, the EMT to promote even greater stemness and malignant behavior in breast cancer cells [19,20]. Our experimental evaluations confirmed the significant differential expression of five genes of the above-noted panel, including *CCNB1*, *CCNB2*, *PTTG1*, *RACGAP1*, and *UBE2C* (EGT gene cluster), in breast tumor samples compared to healthy tissue. These genes were also identified as key players in the networks of fetal male gonad PGCs by our bioinformatics analysis, suggesting a link between the expression of these genes and the EGT in breast carcinoma. In addition, we found that the expression of EGT gene clusters varied according to the age, stage, grade, and receptor status of the patients, indicating that these genes may have different roles and impacts in different subtypes and stages of breast cancer. However, further observations are required to clarify the biological role of this EGT gene cluster in the development of breast carcinoma.

The B-type cyclins, *CCNB1* (*Cyclin B1*) and *CCNB2* (*Cyclin B2*), are integral as regulators of CDK1 (a protein kinase) during mitosis. These cyclins form complexes with CDK1, known as maturation-promoting factors (MPFs), enhancing its catalytic activity and initiating mitosis and the breakdown of the nuclear envelope [21,22]. During the PGC and germline development, the *CCNB1* and *CCNB2* have distinctive roles in both mitosis and meiosis. It has been shown that the *CCNB1* is vital for the G2-M transition in mitosis, while CCNB2’s function is more redundant. In contrast, during meiosis, both *CCNB1* and *CCNB2* are necessary for the entry and exit of the M phase, as well as for chromosome formation and separation. Their subcellular localization and expression patterns differ in gonadal germ cells: *CCNB1* mainly associates with microtubules, while *CCNB2* localizes primarily to the Golgi apparatus. The *CCNB1* is expressed in both male and female germ cells, but *CCNB2* is more abundant in male germ cells [23,24]. It has been shown that the aberrant development of PGCs and testicular germ cell tumors can result from the disruption in *CCNB1* and *CCNB2* genes [25]. The *PTTG1* also is a pituitary tumor-transforming gene that encodes a protein that acts as a transcription and a securing factor. This factor is implicated in enhancing cell proliferation, inhibiting apoptosis, inducing angiogenesis, and affecting gene expression [26]. The overexpression of *PTTG1* may impact the stability of chromosomes and the fidelity of chromosome segregation during cell division. *PTTG1* correlates with the expression of several genes involved in mitosis and cytokinesis regulation, such as kinesin family members (*KIFC1* and *KIF2C*), *cell division cycle 20* (*CDC20*), *CCNB1*, and aurora *kinase B* (*AURKB*). These genes are responsible for the chromosome movement and alignment, activation, and inactivation of *cyclin B/CDK1* and the formation and cleavage of the midbody during cell division [27]. The *RACGAP1* is a gene that encodes for a protein that regulates cytokinesis and the cell polarity in various cell types, including PGCs and male germ cells. *RACGAP1* expression in the PGCs is required for their migration, survival, and proliferation in the gonadal ridges [28,29]. *RACGAP1* seems to be essential for the completion of cytokinesis and the formation of individual spermatids during male meiosis [30]. It has been shown that the *RACGAP1* expression is associated with the formation of the acrosome and the tail of the spermatozoa, which are crucial for their motility and fertilization ability [31]. Furthermore, *UBE2C*, the ubiquitin-conjugating enzyme E2 C gene, plays a role in degrading cell cycle regulators and activating the NF-kappa pathway [32,33]. The *UBE2C* is also involved in male PGC and germline stem cell development, which is crucial for maintaining genome stability in PGCs by resolving transcription–replication conflicts and protecting common fragile sites [34]. Furthermore, *UBE2C* is a part of a pluripotency cycle that regulates germ cell specification and differentiation [35].

Prior clinical and observational studies have demonstrated a positive correlation between the EGT gene cluster and a poor prognosis as well as therapy resistance in patients with breast cancer [36,37,38]. Some studies have reported the role of *CCNB1*, *CCNB2*, *PTTG1*, *RACGAP1*, and *UBE2C* in cell cycle regulation, aggressive tumor behavior, a poor prognosis, resistance to therapy, and cancer stem cell regulation in breast cancer. *CCNB1* and *CCNB2* are frequently overexpressed in breast cancer, especially in the HER2-positive subtype. A high expression of these genes is correlated with lymph–vascular invasion, hormonal receptor negativity, and a shorter survival [39]. *PTTG1* is also overexpressed in breast tumors and breast cancer stem cells and is associated with a high tumor grade, lymphatic metastasis, and poor outcomes of breast cancer cases. The induction of breast cancer cells’ EMT, stemness, and also drug resistance are the other related actions of the *PTTG1* [40,41]. *RACGAP1* overexpression in breast tumors is associated with a high tumor grade, HER2 positivity, and a poor prognosis. *RACGAP1* regulates Rho GTPases, which modulate cytoskeleton dynamics, cell migration, and invasion in cancer. *RACGAP1* also enhances the self-renewal, tumorigenicity, and metastatic potential of breast cancer stem cells [42]. Ultimately, *UBE2C* in breast cancer is associated with a high tumor grade, hormonal receptor negativity, HER2 positivity, and a poor survival. *UBE2C* confers resistance to chemotherapy and targeted therapy in breast cancer cells [38,43]. Considering the role of the EGT genes in both germline and cancer cells, it is suggested that only a specific population of the breast tumor cells could express them.

Based on the findings from our experimental study on the patients’ tumor samples, we further performed an scRNA-seq analysis of the GSE180286 breast tumor dataset to characterize the tumor microenvironment and the cellular heterogeneity within the tumor. We identified 14 distinct clusters of cells, each with unique transcriptomic signatures of EGT genes and cell cycle phases, representing different cellular states within the tumor. Among these clusters, we focused on the breast tumor stromal cells, which exhibited a distinct expression profile, marked by the expression of genes associated with germline cells. The breast tumor stromal cells are a heterogeneous population of cells that reside in the tumor microenvironment and interact with tumor cells and other components of the stroma [44,45]. Stromal cells have been shown to influence various aspects of tumor biology, such as angiogenesis, inflammation, immune evasion, invasion, metastasis, and therapy resistance [45,46]. It has been shown that the stromal cells in solid tumors could exhibit the stemness characteristics [47]. Our study suggests that tumor stromal cells may exploit the mechanisms of PGC development to acquire germline traits and stemness, thereby enhancing their malignant potential. This hypothesis is supported by the observation that the genes we have identified as key players in the networks of fetal male gonad PGCs are also involved in processes such as proliferation, mitosis, and cell division. These genes include *FEN1*, *RACGAP1*, *MCM4*, *CCNB1*, *CCNB2*, *PTTG1*, and *UBE2C*. The overexpression of some of the above-noted genes in breast cancer and their correlation with a poor prognosis and resistance to therapy has previously been proven [36,37,38].

Furthermore, our pseudotime analysis and clustering results (Figure 6) revealed that the seven key EGT genes identified in this study were predominantly expressed within the BC stromal cell cluster, which was spatially positioned in close proximity to epithelial lineages, including stratified, luminal, and progenitor cells (Figure 6A,B). The box plot generated by the Destiny package further illustrated the distribution of cellular states along a developmental continuum, indicating that the stromal population occupies a biologically relevant position within the broader trajectory of cell maturation (Figure 6C). A detailed evaluation of the inferred pseudotime trajectories across epithelial and stromal clusters suggested that stromal cells with a high expression of EGT-related genes are not likely to have arisen from a stem-like or undifferentiated origin. Instead, they may have been generated through a transitional process originating from differentiated breast cancer epithelial cells. These findings support the potential existence of a cellular trajectory from luminal epithelial states toward stratified epithelial phenotypes, ultimately culminating in the emergence of germline-like tumor cells within the epithelial–stromal compartment of the breast tumor microenvironment (Figure 6A,B).

In summary, this study introduces the EGT as a possible developmental mechanism in breast carcinoma. Mechanistically, it could be proposed that a sequential reprogramming process may trigger chromatin remodeling, DNA hypomethylation, and the reactivation of germline transcription factors, ultimately leading to the aberrant expression of typically silenced testis-restricted genes. Such a transition could confer an increased stemness, immune evasion capacity, and therapeutic resistance upon tumor cells. Further research is needed to validate and explore this mechanism and to identify potential biomarkers and therapeutic targets for breast cancer. Our study also has some limitations, including its reliance on a single scRNA-seq dataset, potential differences in gene expression between bulk and single-cell levels, and a lack of investigation into the functional roles of the 11 genes in different cell clusters. Further research is necessary to functionally examine the role of EGT genes in the development of breast carcinoma cells.

## 4. Materials and Methods

### 4.1. Identify the Germline-Associated Genes in Breast Tumors

#### 4.1.1. Bulk mRNA Expression Analysis

We utilized the Gene Expression Omnibus (GEO) database, available at https://www.ncbi.nlm.nih.gov/gds (accessed on 10 March 2021), to access the microarray analysis data of normal and cancerous human mammary gland samples. And subsequently two GSE29044 and GSE42568 studies were included. Our analytical approach involved the GEO2R online tool, reachable at https://www.ncbi.nlm.nih.gov/geo/geo2r/ (accessed on 20 January 2021), which provides robust quality control and comparative analysis capabilities. Differential gene expression between malignant and normal tissues was visualized using the VolcaNoseR system, available at https://huygens.science.uva.nl/VolcaNoseR/ (accessed on 10 March 2021), filtering mRNAs with a fold change (Fc) ≥ 1 and a significance threshold of a *p*-value (PV) ≤ 0.05. Subsequently, common differentially expressed mRNAs and biological process enrichment were identified using FunRich (version 3.1.3), enabling a focused analysis of transcripts relevant to breast cancer. This methodical approach provided detailed insight into the mRNA expression profile in breast cancer, contributing to our understanding of its molecular underpinnings. 

#### 4.1.2. Enrichment Analysis

Comprehensive enrichment analysis of the upregulated genes was employed using the EnrichR system, accessible at https://maayanlab.cloud/Enrichr/ (accessed on 5 May 2021). Utilizing a set of 455 common genes identified from our initial expression analysis of datasets GSE29044 and GSE42568, we focused on cell type enrichment, WebCSEA: https://bioinfo.uth.edu/webcsea/ (accessed on 15 July 2021). Additionally, we employed Enrichr to delve into the Reactome pathway enrichment and associated biological processes. For the visualization of our enrichment analysis, we utilized the ggplot2 package within RStudio (Version 2022.7.1). 

#### 4.1.3. Network Analysis and Identification of Target mRNA

To identify key involved genes, we used protein–protein interaction (PPI) networks within gene sets related to the fetal germ gonad area, and we utilized the STRING database, https://string-db.org/ (accessed on 20 July 2021). For the visualization of the ultimate complex networks, we employed Cytoscape software (version 3.8.2), which facilitated the creation of detailed, interpretable graphical representations.

To analyze the gene expression levels of our targeted genes, we utilized the “The Cancer Genome Atlas” (TCGA) database, accessible at https://ualcan.path.uab.edu/analysis.html (accessed on 23 July 2021), comparing their expression in breast tumor tissues versus normal tissues. 

### 4.2. The Experimental Validation of the Candidate Gene Expression in Breast Tumor Samples

#### 4.2.1. Ethical Considerations and Biopsy Procedures

The experimental phase of this study involved a cohort of 28 female participants who had recently been diagnosed with breast carcinoma. This study took place from January 2021 to April 2023 at the Cancer Institute of Iran, affiliated with Tehran University of Medical Sciences (Tehran, Iran). Tumor tissue samples were collected by the Iran National Tumor Bank at the Cancer Institute of Iran during that period of time. All participants, aged 20 or above, provided their written informed consent prior to the procedure. They underwent ultrasound-guided biopsies with a 14-gauge core needle. A pathologist then conducted tumor histopathological examinations using hematoxylin and eosin (H&E) and immunohistochemistry (IHC) staining. All methods were performed in accordance with the relevant guidelines and regulations and approved by the Institutional Review Board of Tehran University of Medical Sciences, Tehran, Iran (ethical code: IR.TUMS.IKHC.REC.1399.384). The demographic and clinicopathological details of the patient are presented in Figure 4.

#### 4.2.2. SYBR Green Quantitative PCR (qPCR) Techniques

Total RNA was extracted from the tissue samples using the RNA isolation kit (Qiagen, Hilden, Germany; Cat. No. 74534). The isolated RNA was then converted into cDNA using the cDNA synthesis kit (Takara Bio, Shiga, Japan; Cat. No. RR037A). We assessed the expression of targeted mRNAs via quantitative real-time PCR (qRT-PCR), employing SYBR Green Real-Time PCR Master Mixes (Thermo Fisher Scientific, Waltham, MA, USA; Cat. No. 4309155) and conducting the analysis on an ABI Prism 7900HT system in triplicate. For normalization, GAPDH served as the internal control gene, and the ΔΔCt method was used to normalize expression levels. We utilized RStudio, along with the ggplot2 package, for graphical representation of mRNA expression data. The primers were designed using Primer3 software (version 2.5.0), a standard in bioinformatic primer design, ensuring sequence specificity and optimal annealing characteristics. Their sequences were validated for specificity using the NCBI BLAST database, accessible at https://blast.ncbi.nlm.nih.gov/Blast.cgi (accessed on 15 March 2022). Detailed sequences are available in Table 1.

### 4.3. Cellular Clustering of Breast Tumor Cells and Target Cell Detection

In this study, scRNA-seq was conducted on breast cancer cells, utilizing the GSE180286 dataset’s count matrix. Raw count matrices from four breast cancer samples were imported into R and processed as individual Seurat objects using Seurat v4.3.0, available at https://satijalab.org/seurat/ (accessed on 2 November 2023). Quality control filtering was applied per sample: cells with low gene counts, extremely high feature counts, or elevated mitochondrial gene expression were excluded. For instance, Seurat1 was filtered using nFeature_RNA > 400 and < 5000 and percent.mt < 60, while other samples were filtered using optimized cutoffs ranging from 200 to 5800 features and <45–60% mitochondrial content. Filtered Seurat objects were merged into a single object for downstream analysis. Normalization was carried out using the SCTransform method with mitochondrial content regressed out. Highly variable genes were identified using the “vst” method. Principal component analysis (PCA) was performed on the top 20 components. UMAP was then used for non-linear dimensionality reduction (dims = 1:10), with default neighbor and distance settings. Clustering was performed using the Louvain algorithm at a resolution of 0.5.

Cluster annotation was performed using a two-step strategy. First, we identified cluster-specific marker genes using FindAllMarkers (Seurat package, Version 4.3.0) with the following cutoffs: adjusted *p*-value < 0.01 and log2 fold change > 1. Second, these markers were subjected to cell type enrichment analysis using WebCSEA, https://bioinfo.uth.edu/webcsea/ (accessed on 15 November 2023) a phenotype-based cell annotation platform. The most enriched phenotype profiles were then used to assign putative cell type identities to each cluster.

For pseudotime trajectory analysis in our Seurat dataset, we employed the Destiny package, https://bioconductor.org/packages/release/bioc/html/destiny.html (accessed on 21 November 2023), and Monocle3, https://cole-trapnell-lab.github.io/monocle3/ (accessed on 21 November 2023). Briefly, the integrated Seurat object was converted into a Monocle3 cell_data_set. Trajectories were constructed using learn_graph, and cells were ordered with order_cells along pseudotime, using UMAP-based root cells. In parallel, the Destiny package was used to generate diffusion maps, supporting the pseudo-temporal ordering and revealing gradual transcriptional changes. The resulting trajectories reflect a dynamic continuum of cell states, potentially indicative of developmental plasticity and germline-like programs within specific tumor compartments. 

### 4.4. Statistical Methodology

The data were statistically processed and presented as mean ± standard deviation. This analysis was performed using SPSS (Version 26.0; IBM Corp., Armonk, NY, USA). For mean comparison across different groups, an ANOVA test was applied, complemented by a Tukey post hoc test for detailed group analysis. A *p*-value < 0.05 was considered statistically significant in all analyses, and genes with a log2 fold change ≥1 and adjusted *p*-value ≤ 0.05 were selected as significantly deregulated.

## 5. Conclusions

Breast cancer, characterized by its complexity and heterogeneity, presents substantial challenges in diagnosis, prognosis, and treatment. This study introduced a novel concept, the “epithelial-to-germline transition,” elucidating the reactivation of testis cancer antigens and the acquisition of germline characteristics by carcinoma cells in breast tumor development. Through integrative analyses of transcriptomic datasets, we identified a germline-associated molecular signature, with *CCNB1*, *CCNB2*, *PTTG1*, *RACGAP1*, and *UBE2C* emerging as core EGT gene clusters markedly upregulated in breast tumors. The experimental qRT-PCR validation confirmed their overexpression in clinical breast cancer samples, and single-cell transcriptomic profiling pinpointed breast tumor stromal cells as a key population acquiring germline-like features. Collectively, these findings unveil a plausible mechanistic link between germ cell transition pathways and breast cancer progression, highlighting the EGT as a potential driver of phenotypic plasticity and tumor heterogeneity. Further investigations using larger single-cell datasets and functional perturbation studies are required to evaluate the clinical utility of EGT genes as biomarkers or therapeutic targets.

## Figures and Tables

**Table 1:** Forward and reverse primer sequences for human mRNAs.

No	Gene	Sequence (5′ → 3′)		Amplicon Size
1	UBE2C	F	AGTGGCTACCCTTACAATGCG	77
		R	TTACCCTGGGTGTCCACGTT	
2	RACGAP1	F	TGCACGTAATCAGGTGGATGT	81
		R	TGAATCTGTCGTTCCAGCTTTT	
3	PTTG1	F	ACCCGTGTGGTTGCTAAGG	90
		R	ACGTGGTGTTGAAACTTGAGAT	
4	MCM4	F	GACGTAGAGGCGAGGATTCC	182
		R	GCTGGGAGTGCCGTATGTC	
5	FEN1	F	CACCTGATGGGCATGTTCTAC	102
		R	CTCGCCTGACTTGAGCTGT	
6	CCNB2	F	TGCTCTGCAAAATCGAGGACA	180
		R	GCCAATCCACTAGGATGGCA	
7	CCNB1	F	AATAAGGCGAAGATCAACATGGC	111
		R	TTTGTTACCAATGTCCCCAAGAG	
8	GAPDH	F	CTGGGCTACACTGAGCACC	101
		R	AAGTGGTCGTTGAGGGCAATG	

## Data Availability

All the processed data and analysis scripts are available from the corresponding author upon reasonable request.

## Supplementary Materials

The following supporting information can be downloaded at https://www.mdpi.com/article/10.3390/ijms26188958/s1.

## Abbreviations

The following abbreviations are used in this manuscript:

BCSCs	Breast Cancer Stem Cells
CCNB	Cyclin B
CHEK1	Checkpoint Kinase 1
CTAs	Cancer–Testis Antigens
EMT	Epithelial-to-Mesenchymal Transition
EGT	Epithelial-to-Germline Transition
ER	Estrogen Receptor
FEN1	Flap Structure-Specific Endonuclease 1
GEO	Gene Expression Omnibus
GSCs	Germline Stem Cells
Hedgehog	Signaling Pathway
HER2	Human Epidermal Growth Factor Receptor 2
MCM4	Minichromosome Maintenance Complex Component 4
Notch	Signaling Pathway
PGCs	Primordial Germ Cells
Piwil2	Piwi-Like RNA-Mediated Gene Silencing 2
PPI	Protein–Protein Interaction
PR	Progesterone Receptor
PTTG1	Pituitary Tumor Transforming Gene 1
qPCR	Quantitative Polymerase Chain Reaction
scRNA-seq	Single-Cell RNA Sequencing
TCAs	Testis Cancer Antigens
TCGA	The Cancer Genome Atlas
TGF-β	Transforming Growth Factor Beta
UBE2C	Ubiquitin-Conjugating Enzyme E2 C
UMAP	Uniform Manifold Approximation and Projection
